# Persistence of diet-induced obesity despite access to voluntary activity in mice lacking sarcolipin

**DOI:** 10.14814/phy2.12549

**Published:** 2015-09-23

**Authors:** Daniel Gamu, Anton Trinh, Eric Bombardier, A Russell Tupling

**Affiliations:** 1 Department of Kinesiology, University of WaterlooWaterloo, Ontario, Canada

**Keywords:** Obesity, sarcolipin, SERCA, skeletal muscle, voluntary wheel running

## Abstract

Several rodent models of obesity have been shown to develop excessive adiposity only when voluntary cage ambulation is restricted. We have previously shown that mice lacking the sarco(endo)plasmic reticulum Ca^2+^-ATPase pump regulatory protein sarcolipin (*Sln*^*–/–*^), an uncoupler of Ca^2+^ uptake, develop excessive diet-induced obesity under standard housing conditions. However, it is unclear whether this phenotype is due, in part, to the sedentary housing environment in which these animals are kept. To address this, we allowed wild-type and *Sln*^*–/–*^ animals ad libitum access to voluntary wheel running while consuming a standard chow or high-fat diet for 8 weeks. During this period, wheel revolutions were monitored along with weekly mass gain. Postdiet glucose tolerance and visceral adiposity were also taken. The volume of wheel running completed was similar between genotype, regardless of diet. Although voluntary activity reduced mass gain relative to sedentary controls within each diet (*P < *0.05), visceral adiposity was surprisingly unaltered with activity. However, *Sln*^*–/–*^ mice developed excessive obesity (*P *<* *0.05) and glucose intolerance (*P < *0.05) with high-fat feeding relative to wild-type controls. These findings indicate that the excessive diet-induced obese phenotype previously observed in *Sln*^*–/–*^ mice is not the result of severely restricted daily ambulation, but in fact the inability to recruit uncoupling of the Ca^2+^-ATPase pump.

## Introduction

Obesity has reached epidemic proportions globally, and as such, an understanding of its physiological contributions is of extreme interest to the field. At its core, obesity represents a state of chronic energy imbalance, in which cumulative energy intake exceeds that of expenditure, leading to the excessive storage of lipid in both adipose and nonadipose tissue. While current treatments to reduce body mass aim to limit energy intake/absorption, promoting increased energy expenditure by targeting basal metabolic rate, and/or that expended through physical activity may represent an efficacious alternative.

Skeletal muscle comprises ˜40% of adult body mass and contributes ˜20–30% of daily energy expenditure (Rolfe and Brown 1997); thus, it represents a quantitatively significant energy sink. Several important energy-dependent reactions exist within skeletal muscle including that required to regulate cytosolic [Ca^2+^] (Rolfe and Brown 1997), a process mediated by the sarco(endo)plasmic reticulum (SR) Ca^2+^-ATPase (SERCA). SERCAs are 110 kDa integral membrane proteins responsible for maintaining cytosolic [Ca^2+^] in the nanomolar range and initiating muscular relaxation through the adenosine triphosphate (ATP)-dependent translocation of Ca^2+^ into the SR lumen against a concentration gradient (Toyoshima et al. 2000). In mice, SERCA activity can account for up to 48% of resting energy expenditure of slow- and fast-twitch skeletal muscle (Chinet et al. 1992; Decrouy et al. 1993; Dulloo et al. 1994; Smith et al. 2013). This proportion can be further modified by the SERCA regulatory protein sarcolipin (SLN) (Bombardier et al. 2013b).

Sarcolipin is a small 31 amino acid proteolipid expressed abundantly in cardiac atria (Bhupathy et al. 2007) and to varying degrees across skeletal muscle fibers (Fajardo et al. 2013). When bound to SERCA, SLN functions by reducing SERCA’s apparent Ca^2+^ affinity (Asahi et al. 2003). Several in vitro (Smith et al. 2002; Mall et al. 2006) and a recent physiological study (Bombardier et al. 2013b) have shown that SLN can uncouple Ca^2+^ transport from ATP hydrolysis, increasing the energy demand by SERCA to pump Ca^2+^. SLN can promote SERCA inefficiency across a range of metabolic rates (Gamu et al. 2014), including during states of increased spontaneous cage activity, forced treadmill running (Bombardier et al. 2013a), and during acute cold-induced shivering (Bal et al. 2012). Mice lacking SLN (*Sln*^*–/–*^) are prone to excessive diet-induced obesity and glucose intolerance (Bal et al. 2012; Bombardier et al. 2013a) due to increased SERCA efficiency. Furthermore, skeletal muscle SLN expression increases three- to four-fold in response to high-fat feeding (Bal et al. 2012; Bombardier et al. 2013a), indicating that the capacity to uncouple SERCA can increase in an adaptive manner. However, it is not clear whether the obesogenic effect of SLN ablation is unique to the housing environment of these animals, specifically, the restricted access to physical activity.

As is standard in many studies of diet-induced obesity, rodents typically do not have access to voluntary running wheels, severely restricting daily ambulation. This simple fact may complicate the interpretation of metabolic phenotypes associated with transgenic models, as it makes unclear whether the observed phenotype is the direct result of genetic manipulation or that of an interaction with a severely sedentary environment (Booth and Laye 2009). This is exemplified by melanocortin-4 receptor (MC4R) knockout (KO) mice (Huszar et al. 1997; Haskell-Luevano et al. 2009) and OLETF rats (Morris et al. 1985; Rector et al. 2008a,b). Both of these rodent models develop an obese phenotype when sedentary (Morris et al. 1985; Huszar et al. 1997; Rector et al. 2008a); however, simply allowing these animals access to voluntary running wheels normalizes the obesity phenotype caused by gene mutation (Rector et al. 2008a,b; Haskell-Luevano et al. 2009). Thus, in certain models, voluntary activity can compensate for a genetic propensity for weight gain. Additionally, cessation of voluntary activity can have immediate biochemical effects associated with metabolic dysfunction in rodents, including reduced insulin-stimulated skeletal muscle glucose uptake (Kump and Booth 2005a) and fatty acid oxidation (Laye et al. 2009), increased visceral adipose tissue triacylglycerol synthesis, and increased visceral adiposity (Kump and Booth 2005b). The above studies demonstrate that voluntary physical activity optimizes metabolic function and can have a pronounced impact on energy balance.

The objective of this study was to determine whether providing *Sln*^*–/–*^ mice access to voluntary running wheels could mitigate or abolish the obesogenic effect of SLN ablation in response to high-fat feeding. Given that SLN can increase the energy consumed by SERCA across a range of metabolic rates (Gamu et al. 2014), we hypothesized that an excessively obese phenotype would persist in *Sln*^*–/–*^ mice, despite having access to voluntary running wheels during a high-fat diet (HFD).

## Materials and Methods

### Experimental animals

Generation of *Sln*^*–/–*^ animals has been described previously (Babu et al. 2007). Generation of *Sln*^*–/–*^ and wild-type (WT) littermates was achieved through heterozygous breeding (i.e., *Sln*^*+/–*^ X *Sln*^*+/–*^). Prior to experimentation, mice were group housed at room temperature (˜22°C) under a reverse 12:12-h light/dark cycle and given ad libitum access to water and standard rodent chow (22/5 Rodent Diet 8640; Harland-Tekland, Madison, WI). At ˜4 weeks of age, ear clippings were taken from experimental animals and genotyped according to Tupling et al. (2011). Experiments were conducted on 3- to 4-month-old male *Sln*^*–/–*^ and WT littermate controls (C57Bl/6J). All experiments were approved by the University of Waterloo Animal Care Committee and carried out in accordance with the Canadian Council on Animal Care.

### Experimental diet, activity, and glucose tolerance

Individually housed *Sln*^*–/–*^ and WT mice were given either standard rodent chow (as above) or a HFD containing 42% kcal from fat (TD 88137; Harlan Teklad, Madison, WI) for a period of 8 weeks. Furthermore, mice of each genotype were randomly assigned to a sedentary (Sed) treatment group, containing locked running wheels within their cage, or an activity group given ad libitum access to voluntary wheel running (VWR). Sample sizes for each chow-fed group were – WT-Sed: 12, *Sln*^*–/–*^-Sed: 7, WT-VWR: 12, *Sln*^*–/–*^-VWR: 8, while those for HFD animals were – WT-Sed: 9, *Sln*^*–/–*^-Sed: 7, WT-VWR: 14, *Sln*^*–/–*^-VWR: 8. Running wheels were equipped with a magnet and counterbalanced, and activity was monitored using magnetic sensors placed above the running wheels in which wheel revolutions were recorded on an electronic counter. Ambulatory activity (i.e., distance travelled) was determined by multiplying wheel revolutions by the running wheel circumference (40 cm) weekly. Body mass was also measured at the beginning of each week over the dietary period.

Following each diet, whole-body glucose tolerance was measured on fasted mice (˜12 h) using an intraperitoneal glucose tolerance test. Predietary measurements were not included in the statistical analysis as a phenotypic difference is only observed following high-fat feeding (Bal et al. 2012; Bombardier et al. 2013a). Additionally, mice given access to VWR had their wheels locked during the fasting period. Venous blood (˜5–10 *μ*L) was sampled from a tail vein and blood glucose was measured using a glucometer (Accu-Chek Aviva; Roche Diagnostics, Mississauga, ON, Canada) at 0, 30, 60, and 120 min following an injection of 20% d-glucose (dose: 1 g/kg body mass).

### Adiposity

Mice were euthanized with an anesthetic overdose (0.65 mg somnitol/kg body mass), after which epididymal and retroperitoneal fat pads were removed and weighed. Visceral adiposity was calculated from the fat pad weights as an adiposity index, defined as ([sum of fat pad mass]/body mass) × 100 (Taylor and Phillips 1996). Additionally, the soleus (SOL) muscle was excised, cleared of connective tissue, and weighed.

### Statistical analysis

Data were presented as mean ± SE. When appropriate, data were analyzed using a one-way analysis of variance (ANOVA) with repeated measures, two-way ANOVA with repeated measures, and a two-way ANOVA with independent samples. Specific mean differences were examined using a Newman–Keuls post hoc test. Statistical significance was considered at *P *<* *0.05.

## Results

### VWR activity, mass gain, and adiposity

**Figure 1:**
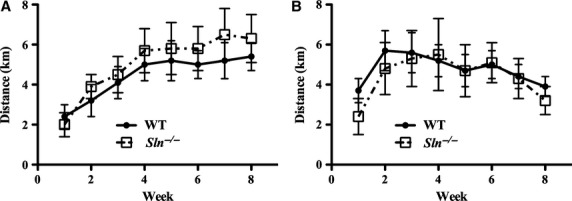
Average daily voluntary wheel running distance (km) of wild-type (WT) and *Sln*^–/–^ mice during 8 weeks of (A) chow or (B) high-fat feeding. Voluntary running activity for experimental animals was calculated as described in “Materials and Methods” section. Values displayed are mean ± SE.

Voluntary running activity of chow- and high-fat-fed animals was variable and no differences in average daily activity existed between WT and *Sln*^*–/–*^ mice over the 8-week measurement period, regardless of diet (Fig. 1). Furthermore, no genotype differences in activity were found when measured as total VWR volume (i.e., Student’s *t*-test, independent samples) in chow-fed (*P *=* *0.991; 282.7 ± 46.7 km vs. 281.9 ± 50.6 km, WT vs. *Sln*^*–/–*^, respectively) or high-fat-fed animals (*P *=* *0.764; 257.8 ± 36.2 km vs. 238.9 ± 52.4 km, WT vs. *Sln*^*–/–*^, respectively).

**Figure 2:**
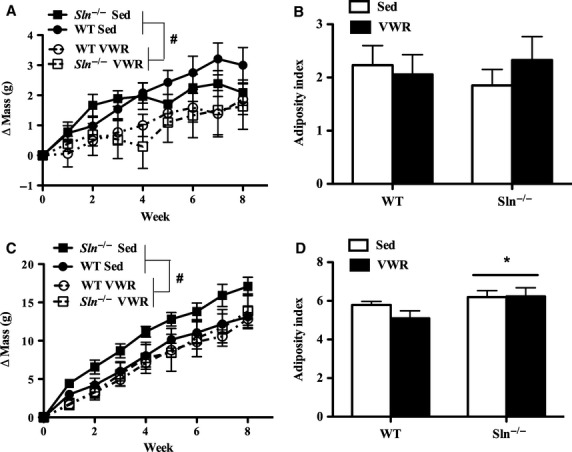
Average weekly mass gain (g) of sedentary (Sed) and voluntary wheel running (VWR) wild-type (WT) and *Sln*^*–/–*^ mice during 8 weeks of (A) chow or (C) high-fat feeding. Visceral adiposity of (B) chow- or (D) high-fat-fed animals. #Significant main effect (*P *<* *0.05) of activity status (VWR < Sed). *Significant main effect (*P *<* *0.05) of genotype (*Sln*^*–/–*^ > WT). Values displayed are mean ± SE.

Next, dietary mass gain was measured within each dietary treatment in response to VWR. As expected, mice given access to VWR gained less body mass relative to Sed counterparts (main effect of activity status: *P *<* *0.05) on both the chow (Fig. 2A) and HFD (Fig. 2C). Surprisingly, visceral adiposity of chow-fed animals was unaffected by VWR access (Fig. 2B), nor were there any differences in adiposity as a result of SLN ablation. Additionally, absolute SOL and fat pad mass (Table 1) were unaltered in chow-fed animals. However, a phenotypic difference did emerge in response to the HFD (Fig. 2D), with *Sln*^*–/–*^ mice becoming significantly more obese than WT animals as indicated by the higher visceral adiposity index regardless of activity status (main effect of genotype: *P < *0.05). Similar to chow-fed animals, VWR itself had no impact on visceral adiposity relative to Sed counterparts. The increase in adiposity with SLN ablation was not associated with any change in skeletal muscle mass, as noted by similar SOL mass between treatment groups (Table 2). Additionally, absolute masses of the epididymal and retroperitoneal fat pads (Table 2) were greater with SLN ablation, regardless of activity status (main effect of genotype: *P < *0.05). Together, this suggests that the change in adiposity of *Sln*^–/–^ mice is specific to the expansion of adipose tissue and not a decrease in lean mass.

**Table 1:** Skeletal muscle and fad pad mass (mg) of chow-fed Sed and VWR animals

	WT		*Sln*^*–/–*^	
Mass (mg)	Sed	VWR	Sed	VWR
SOL	8.4 ± 0.4	8.0 ± 0.4	7.9 ± 0.7	7.6 ± 0.7
Epididymal	259.2 ± 34.1	225.8 ± 58.1	176.4 ± 50.7	273.9 ± 56.4
Retroperitoneal	123.2 ± 34.2	112.8 ± 46.1	112.6 ± 22.8	142.5 ± 37.5

Values displayed are mean ± SE. Sed, sedentary; VWR, voluntary wheel running; WT, wild type; SOL, soleus.

**Table 2:** Skeletal muscle and fad pad mass (mg) of Sed and VWR animals following 8 weeks of high-fat feeding

	WT		*Sln*^*–/–*^	
Mass (mg)	Sed	VWR	Sed	VWR
SOL	8.9 ± 0.5	9.9 ± 0.6	10.0 ± 1.1	9.4 ± 0.4
Epididymal	738.6 ± 34.1	679.8 ± 58.1	835.6 ± 50.7	905.9 ± 116.1
Retroperitoneal	460.4 ± 34.2	419.4 ± 46.1	585.9 ± 37.5	549.3 ± 82.0

Values displayed are mean ± SE. A significant main effect of genotype (*P *<* *0.05) existed for epididymal (*Sln*^*–/–*^ > WT) and retroperitoneal (*Sln*^*–/–*^ > WT) fat pad mass. Sed, sedentary; VWR, voluntary wheel running; WT, wild type; SOL, soleus.

### Whole-body glucose tolerance

**Figure 3:**
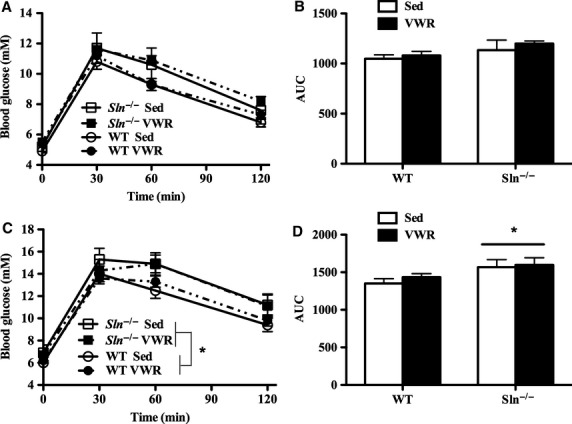
Postdiet glucose tolerance (mmol/L) of wild-type (WT) and *Sln*^–/–^ mice that were sedentary (Sed) or given access to voluntary wheel running (VWR) during 8 weeks of (A) chow or (C) high-fat feeding. Postdiet glucose tolerance expressed as area under the curve (AUC) of (B) chow- or (D) high-fat-fed animals. *Significant main effect (*P *<* *0.05) of genotype (*Sln*^*–/–*^ > WT). Values displayed are mean ± SE.

Previous studies have shown that SLN ablation results in excessive diet-induced glucose intolerance under sedentary housing conditions (Bal et al. 2012; Bombardier et al. 2013a). Thus, it was of interest to determine if this phenotype persisted when animals were given access to increased ambulatory activity. Glucose tolerance was unaffected by both SLN ablation and access to VWR under chow-fed conditions (Fig. 3A and B). Although VWR did not alter glucose tolerance relative to sedentary controls in either genotype following the 8-week HFD, *Sln*^*–/–*^ animals were significantly more glucose intolerant than WT animals regardless of activity status (main effect of genotype: *P *<* *0.05), as indicated by the higher blood glucose concentration (Fig. 3C) and area under the glucose curve (Fig. 3D).

## Discussion

Recently, genetic ablation of SLN has been shown to result in excessive diet-induced obesity under standard housing conditions (i.e., restricted cage ambulation) (Bal et al. 2012; Bombardier et al. 2013a). Several rodent models of obesity have demonstrated that simply giving research animals the ability to increase energy expenditure through voluntary activity can reverse a genetic propensity toward obesity (Rector et al. 2008a,b; Haskell-Luevano et al. 2009), complicating the interpretation of a gene/protein’s role in energy balance. Thus, the objective of the current study was to examine whether ad libitum access to voluntary activity could attenuate the obesogenic phenotype previously observed in *Sln*^*–/–*^ mice when fed a HFD. Surprisingly, VWR had no effect on visceral adiposity of both chow- and high-fat-fed animals. However, as hypothesized the existence of an excessive diet-induced obese phenotype persisted in *Sln*^*–/–*^ animals despite them completing a similar volume of voluntary exercise as WT littermates. Our findings indicate that an extreme sedentary environment is likely not a causative factor in the development of excessive diet-induced obesity in this model, and that the effect of SLN ablation is the prevailing mechanism contributing to this phenotype. In light of these findings, SLN may represent a true “obesity gene” given that lack of an ability to recruit its thermogenic action results in excessive diet-induced adiposity, irrespective of the activity environment.

Rodents have been shown to be extremely active when provided with a means of voluntary activity (Sherwin 1998). In the current study, wheel running activity was similar to previous reports (Lightfoot et al. 2004; Davidson et al. 2006; Meek et al. 2009), although others have reported higher mean running distances for male C57Bl/6J mice (Lerman et al. 1985; Waters et al. 2004; De Bono et al. 2006). Similar to previous studies (Bal et al. 2012; Bombardier et al. 2013a), excessive obesity in *Sln*^*–/–*^ was only present following high-fat feeding, consistent with an inability of these animals to recruit diet-induced uncoupling of SERCA-mediated Ca^2+^ pumping. As expected, VWR was effective at reducing body mass in both chow- and high-fat-fed animals, but surprisingly, visceral adiposity was unaltered under both dietary conditions. It is unclear why discordance between body mass and visceral adiposity existed with VWR in the current study, although it is possible that other adipose depots (e.g., subcutaneous) may have been responsive to the increase in daily ambulation within each diet. Regardless of this, a clear diet-induced obesity phenotype existed with SLN ablation. In agreement with previous findings on sedentary *Sln*^*–/–*^ mice (Bal et al. 2012; Bombardier et al. 2013a), excessive diet-induced obesity was the result of adipose tissue expansion and not a reduction in lean mass, as noted by greater visceral fat pad mass, while skeletal muscle mass was unaltered by genotype or activity status. Consistent with this, an exaggerated impairment in glucose handling mirrored the adiposity changes of high-fat-fed *Sln*^*–/–*^ animals, possibly due to ectopic accumulation of lipid in peripheral tissues. Together these findings indicate that VWR, when coupled with a HFD in this model, is insufficient to reduce visceral obesity and glucose intolerance relative to sedentary conditions. This is likely due to a low caloric demand of VWR relative to the caloric excess of the HFD. Additionally, given that an exaggerated diet-induced obesity phenotype existed in *Sln*^*–/–*^ animals regardless of activity status, the obesogenic effect of SLN ablation observed here and previously (Bal et al. 2012; Bombardier et al. 2013a) is unlikely to be due to a severely sedentary cage environment.

Previous studies demonstrating an impact of VWR on a metabolic phenotype differ from our model in several ways. Unlike MC4R-KO and OLETF models, obesity with SLN ablation is only present during caloric excess, not when animals are fed standard rodent chow (Morris et al. 1985; Huszar et al. 1997; Rector et al. 2008a,b). The lack of an effect of VWR on visceral adiposity in the current study, in both WT and *Sln*^*–/–*^ animals, is likely reflective of the low caloric demand of the voluntary running completed relative to that taken in from high-fat feeding. Additionally, these models have altered signaling of pathways involving regulation of energy intake, and obesity is a function of hyperphagia, whereas the development of obesity in *Sln*^*–/–*^ mice is the result of an inability to recruit diet-induced thermogenesis in response to calorie excess (Bal et al. 2012; Bombardier et al. 2013a).

Several limitations exist with the current study. First, our experimental conditions were carried out at room temperature (˜22°C), which is below thermoneutrality (˜30°C) for mice. Our rationale for conducting the current study below thermoneutral temperatures was to be consistent with our initial study in this model (Bombardier et al. 2013a). It is possible that if these same experiments are done at 30°C that the increase in activity thermogenesis from VWR will result in the predicted reduction in adiposity in both WT and SLN-KO mice given that caloric expenditure from other adaptive mechanisms are less likely to mask this effect at thermoneutrality. Finally, it is unclear what effect VWR has on energy intake in our model system. We have previously shown that 24-h high-fat food intake is unchanged with SLN ablation when sedentary animals are housed in metabolic chambers (Bombardier et al. 2013a). Consistent with this finding, VWR did not impact 24-h food intake when placed in these chambers (data not shown). However, our metabolic system does not accommodate running wheels; thus, food intake in the home cage environment during the 8-week dietary regime may differ from our terminal measurements.

The findings of this current study indicate that increasing energy expenditure through voluntary activity does not attenuate the excessive diet-induced obesity phenotype seen with SLN ablation and confirms that our previous findings on *Sln*^*–/–*^ mice (Bombardier et al. 2013a) are in fact the result of an inability to recruit SLN-mediated uncoupling of SERCA and not the result of severely restricted cage ambulation. In light of these findings, SLN’s contribution to energy expenditure may be indispensible toward body mass regulation, as increasing voluntary activity-induced energy expenditure is insufficient to counter excessive diet-induced adiposity in its absence.

## Conflict of Interest

None declared.
